# Delayed presentation of a snake bite with multiple life threatening complications in third trimester pregnancy successfully managed in resource limited setting: case report

**DOI:** 10.1016/j.xagr.2026.100603

**Published:** 2026-01-10

**Authors:** Leul Endalamaw Demilew, Misganaw Abere Worku, Girmachew Tesfaye Agegnehu, Asnakew Amisalu Mebratu, Gashaw Awoke Haile, Bezawit Alemu Mengist, Wondwosen Mengist Dereje

**Affiliations:** 1 Department of Gynecology and Obstetrics, University of Gondar College of Medicine and Health Sciences, Gondar, Ethiopia (Demilew, Worku, Mebratu, and Mengist); 2 Department of Anesthesiology, University of Gondar College of Medicine and Health Sciences, Critical Care and Pain Medicine, Gondar, Ethiopia (Haile); 3 Department of Neurology, University of Gondar College of Medicine and Health Sciences, Gondar, Ethiopia (Dereje)

**Keywords:** acute kidney injury, antisnake venom (ASV), coagulopathy, pregnancy, snake bite

## Abstract

**Introduction and importance:**

Snakebite envenoming is a potentially life-threatening condition and a common public health problem, particularly in developing countries. Envenomation during pregnancy is rare but carries serious consequences for both the mother and the fetus. Antivenom is the primary treatment; however, its unavailability, along with limited healthcare facilities in developing countries, contributes to significant mortality. In the reported case, we present a 35-year-old woman in her third trimester who developed multiple complications from a snakebite and was managed at our institution.

**Case presentation:**

A 35-year-old gravida VI, para V woman was referred from a primary hospital due to the unavailability of an obstetrician, blood products, appropriate diagnostic facilities, and essential medications. She initially presented to the primary hospital with complaints of vaginal bleeding and decreased fetal movement lasting 24 hours. Additionally, the patient reported lightheadedness, easy fatigability, and blurred vision but denied headaches or any abnormal body movements.

**Conclusion:**

Although snakebite is a common public health problem, it is often neglected. As with other neglected tropical diseases, estimating the global morbidity, disability, and mortality caused by snakebite envenoming is challenging. The disease is prevalent among individuals in impoverished agricultural and herding communities in low- and middle-income countries, where access to healthcare is limited and health-seeking behavior may be poor, making this health issue potentially fatal. Snakebite envenomation during pregnancy has severe consequences for both the mother and the fetus. Increasing community awareness, ensuring early admission, close follow-up, and appropriate management through a multidisciplinary approach can improve outcomes for both mother and fetus.

## Introduction

Snakebite envenomation (SBE) is a life-threatening condition caused by the injection of toxins following the bite of a venomous snake. It predominantly affects individuals living in poor, rural communities in tropical and subtropical regions. Approximately 50% to 55% of all snakebites result in envenomation.^1^

Although rare during pregnancy, snakebite has been reported in about 1% of hospital admissions for pregnant women in a large series from India.^2^^,^^3^ Snakebite remains a common and neglected public health problem.^4^

## Case presentation

A 35-year-old gravida VI, para V woman (all previous deliveries were vaginal without pregnancy or delivery-related complications, and all her children are alive) was referred to our tertiary hospital. The gestational age, calculated from a reliable last normal menstrual period (20/05/2024), was 36 weeks. She began antenatal care (ANC) late, at five months of pregnancy, and did not have an early ultrasound evaluation. She attended a total of three ANC visits, during which she received two doses of the tetanus vaccine and was screened for syphilis, HIV, and HBsAg, all of which were nonreactive. Her blood group is O positive. She was also dewormed during her ANC follow-up.

The patient initially presented to the primary hospital with complaints of massive bright red vaginal bleeding and decreased fetal movement for 24 hours. She also reported lightheadedness, easy fatigability, and blurred vision of the same duration. She denied any headache, abnormal body movements, epigastric pain, or jaundice. At the primary hospital, she was resuscitated with one bag of normal saline and underwent laboratory investigations: CBC showed WBC 7.63 × 10^3/µL with 76% neutrophils and 20% lymphocytes; hemoglobin was 5 g/dL; hematocrit 16.3%; MCV 83.5 fL; and platelets 141 × 10^3/µL.

Three days prior to the onset of vaginal bleeding and decreased fetal movement, she sustained a snakebite on her right foot, near the ankle. Following the bite, hot metal was applied to the site as a traditional remedy, but she did not seek medical attention at that time. Immediately after the trauma, she experienced severe pain and swelling around the bite site. On the fourth day postbite, she began to have massive bright red vaginal bleeding, enough to soak her underwear, accompanied by bilateral nasal bleeding. She was then taken to the primary hospital, where she stayed for 24 hours. At that facility, she received two liters of normal saline for resuscitation and tetanus toxoid but did not receive blood transfusions due to the lack of blood products. After stabilization and bleeding subsidence, she was referred to our institution.

Upon arrival at our hospital, she was conscious and alert. Her vital signs were: blood pressure 90/60 mmHg, pulse rate 110 bpm, respiratory rate 21 breaths per minute, and temperature 36°C. Her body mass index (BMI) was 26 kg/m². Physical examination revealed pale conjunctivae, normal respiratory system findings, and obstetric examination showed a term-sized gravid uterus with cephalic presentation, absent fetal heartbeat, and no uterine contractions. On genitourinary examination, the vulva was soaked with blood; the cervix was 1.5 centimeters dilated and 70% effaced, with no active bleeding. Neurologic examination findings were not documented.

Obstetric ultrasound confirmed a singleton intrauterine pregnancy at an average gestational age of 36 weeks, absence of fetal heartbeat, and a fundal posterior placenta.

She was admitted immediately to the labor ward, and a double intravenous line was secured. Laboratory investigations including random blood sugar, CBC, coagulation profile, renal function tests, and liver function tests were performed (Table 1). She was transfused with three units of whole blood, two units of platelets, and two units of fresh frozen plasma (FFP).

**Table 1:** Laboratory investigations over time

Investigation	27/01/2025	29/01/2025	01/02/2025	03/02/2025	05/02/2025	07/02/2025	10/02/2025	13/02/2025	15/02/2025	18/02/2025
CBC										
WBC (*10³/μL)	6.14	6.74	7.3	7.9	10.5	8.1	6.28	7.2	8.9	7.5
Hb (g/dL)	4.8	7.1	8.2	8.7	9.5	9.3	9.5	9.7	9.9	12
HCT (%)	13.4	21	25.1	26.3	28	27.2	26.4	29.6	30.8	36.1
PLT (*10³/μL)	118	135	143	152	168	201	222	207	193	212
Liver Function Tests										
SGOT (U/L)	42	81	80	45	41	39	31	34	29	31
SGPT (U/L)	23	38	50	34	32	30	32	36	31	29
Renal Function Tests										
BUN (mg/dL)	65	67	64	70	26	22	22	20	17	15
SCr (mg/dL)	1.93	1.93	2.94	1.51	1.42	1.26	1.24	1.21	1.17	0.97
Coagulation Profile										
PT (sec)	37.7	27	16	14	13	11	11.2	12	12.7	13.1
aPTT (sec)	100	72	54	42	34	31.2	29	30	27	27.9
INR	3.6	2.4	2.1	1.6	1.4	1.21	1.09	1.1	0.9	1
Serum Electrolytes										
Na⁺ (mmol/L)	137	142	139	135	134	136	137	136.7	142	139
K⁺ (mmol/L)	4.1	3.5	2.79	3.49	3.7	3.76	3.9	3.8	4.2	4.7
Ca²⁺ (mmol/L)	1.23	1.11	1.09	1.11	1.07	1.04	1.08	1.12	1.1	1.07
Cl⁻ (mmol/L)	104	116	116	113	110	114	112	111	110	109

Following consultation with a maternal-fetal medicine specialist, emergency induction of labor was decided. Considering the risk of postpartum hemorrhage, blood products were prepared, an anesthesiologist was consulted, laparotomy instruments were made available, and informed written consent was obtained. Transcervical balloon catheterization was performed, and oxytocin infusion (2.5 IU in 1000 ml normal saline at 20 drops per minute) was started. Labor progress and antepartum hemorrhage were closely monitored using standardized follow-up sheets.

After 12 hours of labor induction overnight, she delivered a 2.7 kg stillborn fetus.

Postdelivery, she developed profuse vaginal bleeding diagnosed as postpartum hemorrhage due to uterine atony. High-dose oxytocin, misoprostol 800 micrograms, intramuscular ergometrine 0.25 mg, and tranexamic acid 1 gram over 10 minutes were administered. Uterine massage and bimanual uterine compression were performed, but bleeding persisted, necessitating transfer to the operating room for laparotomy.

**Figure 1:**
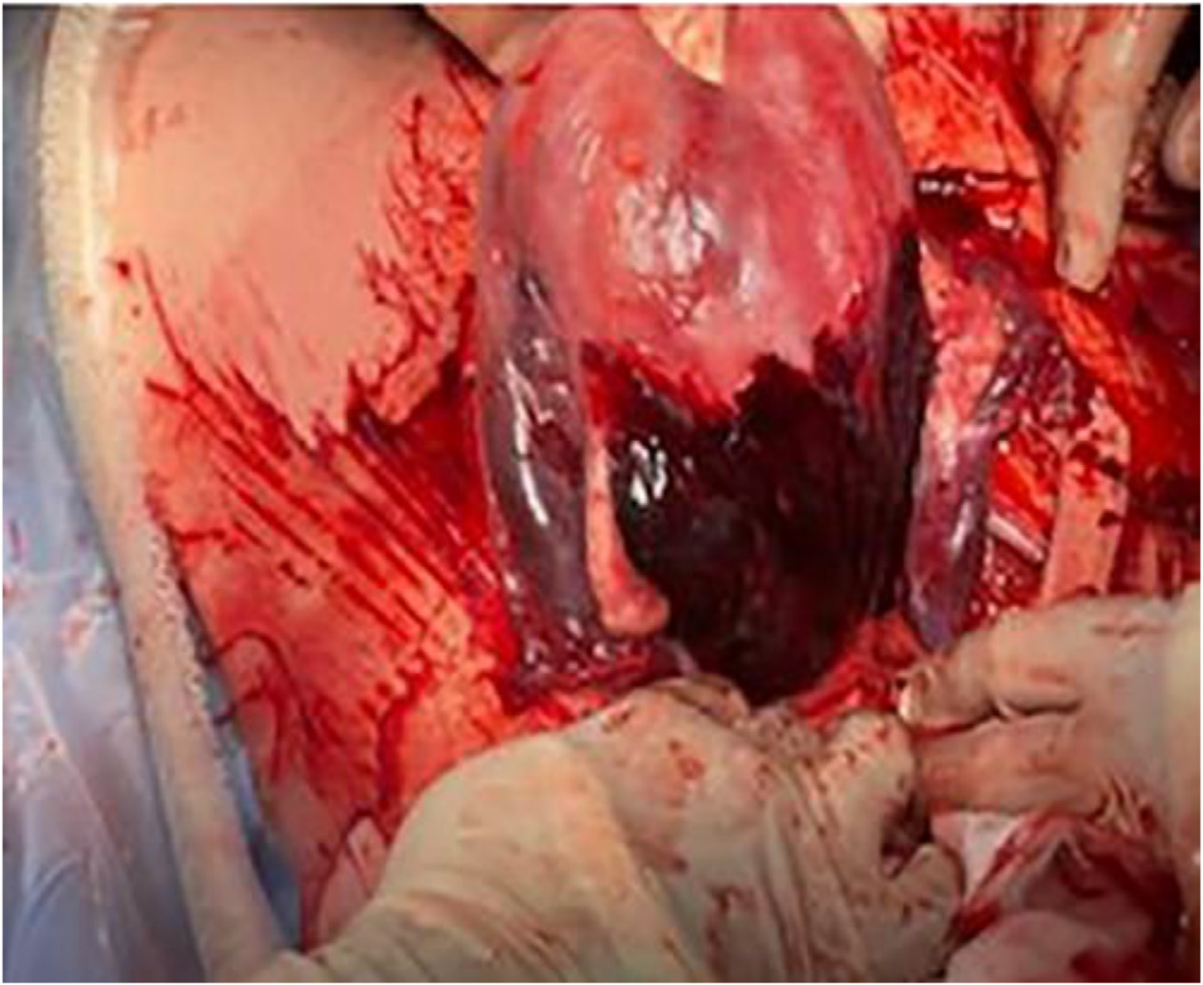
Intraoperative finding dusky and laxed uterus with Couvelaire uterus sign.

During emergency laparotomy, the uterus appeared dusky and lax, with signs of Couvelaire uterus observed (Figure 1). The ovaries and fallopian tubes were intact and healthy. A subtotal (supracervical) hysterectomy was performed, the vaginal cuff was closed, and hemostasis secured. Despite ongoing blood and fluid resuscitation, her blood pressure dropped to 80/50 mmHg, prompting initiation of inotropic support with adrenaline. Intraoperatively, two units of whole blood and two units of platelets were transfused. She left the operating room with blood pressure 98/57 mmHg and pulse rate 113 bpm and was transferred to the intensive care unit (ICU) on an adrenaline drip since we have no maternal ICU.

Demilew et al. Delayed presentation of a snake bite with multiple life threatening complications in third trimester pregnancy successfully managed in resource limited setting. Am J Obstet Gynecol 2025.

In the ICU, she received an additional five units of whole blood, two units of platelets, and six units of FFP. Due to the absence of a toxicology specialist, after consultation with the medical team, antivenom was administered twice (a total of 11 vials). Furthermore, intravenous ceftriaxone 1 gram twice daily and metronidazole 500 mg three times daily were initiated.

On the immediate postoperative day, she developed crepitations and decreased air entry over the bilateral lower thirds of her chest, and type 1 respiratory failure was suspected. Although shock resolved on the first postoperative day, she experienced a single episode of generalized tonic-clonic seizure and was started on phenytoin.

Throughout her ICU stay, she received comprehensive care including deep vein thrombosis prophylaxis and attentive nursing. She was diagnosed with ventilator-associated pneumonia, moderate hypokalemia, and antibiotic-associated diarrhea, all of which were treated successfully. She was extubated on her seventh ICU day. By this time, her disseminated intravascular coagulation (DIC) had improved, and acute kidney injury (AKI) was resolving.

By the end of her ICU stay, she had received a total of 17 units of whole blood, 3 units of packed red blood cells, 13 units of FFP, and 7 units of platelets. On her sixth day postextubation, she was transferred to the ward.

After one week of ward care with wound management and close monitoring, and a total of 21 days of hospital stay and 40 units of blood products transfusion, she was discharged with significant improvement. Her vital signs at discharge were stable: blood pressure 110/70 mmHg, pulse rate 86 bpm, respiratory rate 21 breaths per minute, temperature 36.5°C, and oxygen saturation 96%.

During subsequent outpatient follow-ups, she reported no new complaints and was eventually discharged from care and regional zone journalist visit her and she was advised to inform the community the need of blood donation and early seeking health care when there is snake bite.

## Discussion and conclusions

Snakebite is a major public health concern in developing countries. The estimated annual incidence of snakebite envenomation ranges from 420,000 to 1,841,000 cases, resulting in 20,000 to 150,000 deaths.^5^^,^^6^ In Sub-Saharan Africa, the most venomous snake species belong to the Elapidae and Viperidae families.^7^ Among pregnant women, snakebite accounts for approximately 0.4% to 1.8% of cases and is associated with a maternal mortality rate of 4.2% and a fetal mortality rate ranging from 43% to 58%.^2^^,^^8^

Most snakebites are nonvenomous.^7^ Snakebite envenoming is a potentially life-threatening condition that occurs when a venomous snake injects a mixture of toxins (“venom”) through its bite. Envenoming can also result from venom being sprayed into a person’s eyes by certain snake species capable of spitting venom as a defensive mechanism. Importantly, not all snakebites lead to envenoming; some snakes are nonvenomous, and even venomous snakes may not inject venom with every bite.^9^

Snake venom contains more than 20 different compounds, primarily proteins and polypeptides. The proteins are responsible for nearly all of the venom’s biological and clinical effects.^10^^,^^11^

Pro-coagulant enzymes found in viperid and elapid venoms include digestive hydrolases, phospholipases, thrombin-like pro-coagulants, and kallikrein-like serine proteases, which deplete clotting factors and ultimately lead to consumption coagulopathy. Metalloproteinases contribute to hemorrhage by damaging the endothelial lining of blood vessels, causing both local and systemic bleeding. Additionally, phosphodiesterases interfere with the cardiovascular system, primarily by lowering blood pressure.^10^^,^^11^^,^^12^

Maternal and fetal complications from snakebite envenomation depend on the severity of envenomation.^8^

Several factors influence the severity of clinical manifestations, including the species of snake involved. Consequently, the degree of toxicity following snakebite varies widely. The main venomous snake families Hydrophidae, Elapidae, and Viperidae differ in their appearance, geographic distribution, and venom composition.^13^

The most notorious venomous species, Elapidae and Viperidae, deplete clotting factors leading to consumptive coagulopathy and damage the endothelial lining, resulting in both local and systemic hemorrhage.^7^ The pathophysiological effects of venom during pregnancy are not fully understood. However, venom is thought to cross the placenta and affect the fetus, with foci of necrosis and vascular congestion having been reported.^14^

Zugaib et al. reported that a toxin present in snake venom acts as a coagulative agent. Even in small amounts, this toxin can reach the placental circulation at the decidual-placental cleavage zone and initiate its dissociation.^15^^,^^16^

The most common adverse obstetric events associated with snakebite include vaginal bleeding, intrauterine death (IUD), premature labor, and threatened abortion. Fetal loss is primarily attributed to delayed administration of antivenom (ASV) and several possible mechanisms, including:

1. Direct toxic effect of venom on the fetus.
2. Fetal hypoxia secondary to maternal shock.
3. Venom-induced uterine contractions.
4. Placental bleeding resulting from coagulopathy.^17^

Venomous snakebite during pregnancy is also associated with increased maternal morbidity and mortality, particularly in low-resource settings where delayed presentation is common. It can lead to excessive bleeding, including postpartum hemorrhage. Cases of acute kidney injury requiring dialysis following administration of multiple doses of antivenom after snakebite have also been described.^18^

Snakebite-induced coagulopathy remains the most life-threatening complication, with poor maternal and perinatal outcomes.^19^

Some congenital malformations, such as hydrocephalus and polydactyly, have been reported following snakebite during the first trimester. Other possible outcomes include cleft palate, facial deformities, hepatic and myocardial damage, and embryonic death.

In our patient, the complications included severe placental abruption, acute kidney injury, coagulopathy, shock, and fetal demise.

Evidence to guide the management of this rare obstetric complication remains limited. The definitive treatment for envenomation is the administration of specific antivenom.^12^

Symptoms of envenomation produce characteristic clinical manifestations depending on the species involved, which may be similar or more pronounced during pregnancy due to maternal physiological changes. Pharmacologic management focuses on symptomatic and supportive care, with specific therapy administered when available. The decision to use antidotal therapy (antivenom) must consider the risk of allergic reactions, including Type 1 hypersensitivity (anaphylaxis) and Type 3 hypersensitivity (serum sickness), and the risk–benefit assessment in pregnancy should account for potential adverse effects on the fetus. Antivenoms—currently available for certain snakes, spiders, and scorpions—are generally indicated in cases of^1^ evidence of systemic envenomation or^2^ severe local envenomation. Consultation with a poison center and medical toxicologist is recommended when managing pregnant patients.^20^

In developing countries, limited access to blood products, hematologists, obstetricians, and antivenom often leads to delayed presentation and poorer prognosis. Antivenom remains the definitive treatment, and as reported by Hbib A.G. et al., the primary objective is to save the mother’s life.^17^ There are no absolute contraindications to antivenom, as the benefits of administration outweigh the potential risk of allergic reactions.^18^

Ideally, antivenom should be administered within six hours of the bite; however, it can still be given within 24 hours if necessary.^13^^,^^16^

The maximum duration of antivenom efficacy has not been well studied. In our patient, despite presenting late, antivenom was administered twice after consultation with internists, as no toxicology specialist was available in our setting.

All patients receiving antivenom should be admitted for observation, maintenance antivenom dosing, and repeat laboratory testing until abnormalities resolve. The manufacturer-recommended maintenance dosing consists of two vials of antivenom every six hours for three consecutive doses. The treating physician may adjust the redosing schedule based on the patient’s clinical response and course.^18^

Patients should be closely monitored for any recurrence of signs and symptoms that may require additional antivenom. Clinical deterioration should prompt repeat dosing and timely consultation with a toxinologist or poison control center.^15^ Given the concerns about pregnancy and related risks, envenomated pregnant patients should be observed for longer periods or admitted for continued monitoring and additional treatment as needed.^20^

Antivenom remains the definitive treatment for snakebite envenomation during pregnancy, with indications similar to those in nonpregnant patients. However, its safety in pregnancy has not been evaluated through robust clinical trials.^20^ The currently available evidence suggests that antivenom use is safe in pregnancy and supports the principle that what is beneficial for the mother is also beneficial for the fetus.^21^ Although evidence-based recommendations are limited, management of snakebite envenomation in pregnancy includes supportive care, administration of antivenom in severe cases, anticipation and prompt treatment of anaphylaxis, and close maternal–fetal monitoring during hospitalization.^21^ The authors recommend that care be provided in an intensive care unit, with continuous cardiac and vital-sign monitoring for the mother. Laboratory parameters should be assessed repeatedly to detect any deterioration. In cases with a viable fetus, continuous fetal monitoring should also be employed.

More long-term evaluations of individual cases are encouraged to better characterize the outcomes of specific envenomations during pregnancy and to identify additional strategies beyond standard therapies.^20^

Due to late presentation and multiple lethal complications from the snakebite, our patient was admitted for a total of three weeks and subsequently followed up in the outpatient department for an additional four weeks.

### Conclusions

This case highlights the severe maternal complications that can arise from snakebite envenomation during pregnancy, particularly in resource-limited settings. Delayed presentation, lack of access to antivenom, and limited healthcare infrastructure significantly contributed to the patient's critical condition. Multidisciplinary management, including surgical intervention, intensive care support, and timely blood product administration, was crucial in saving the mother's life. Early recognition, appropriate referral, and availability of essential resources such as antivenom and blood products are vital in improving maternal outcomes. This case underscores the need for increased community awareness and strengthened healthcare systems to manage such complex obstetric emergencies effectively.

## CRediT authorship contribution statement

**Leul Endalamaw Demilew:** Writing – review & editing, Writing – original draft, Visualization, Investigation, Data curation. **Misganaw Abere Worku:** Writing – review & editing, Writing – original draft, Supervision, Investigation. **Girmachew Tesfaye Agegnehu:** Writing – review & editing, Writing – original draft, Supervision, Investigation. **Asnakew Amisalu Mebratu:** Writing – review & editing, Writing – original draft, Supervision, Investigation. **Gashaw Awoke Haile:** Writing – review & editing, Writing – original draft, Supervision, Investigation. **Bezawit Alemu Mengist:** Writing – review & editing, Writing – original draft, Investigation. **Wondwosen Mengist Dereje:** Writing – review & editing, Writing – original draft, Visualization, Validation, Formal analysis, Data curation, Conceptualization.
